# Probability-Based Indoor Positioning Algorithm Using iBeacons

**DOI:** 10.3390/s19235226

**Published:** 2019-11-28

**Authors:** Tianli Wu, Hao Xia, Shuo Liu, Yanyou Qiao

**Affiliations:** 1 Aerospace Information Research Institute, Chinese Academy of Sciences, No.9 Dengzhuang South Road, Haidian District, Beijing 100094, China; wutl@radi.ac.cn (T.W.); liushuo@radi.ac.cn (S.L.); qiaoyy@radi.ac.cn (Y.Q.); 2 University of Chinese Academy of Sciences, No.19(A) Yuquan Road, Shijingshan District, Beijing 100049, China; 3 The Institute of Remote Sensing and Digital Earth, No.20 Datun Road, Chaoyang District, Beijing 100101, China

**Keywords:** indoor positioning, Bluetooth low energy, nearest neighbor, advanced weighted centroid, iBeacon, probability distributions

## Abstract

High-precision indoor positioning is important for modern society. This paper proposes a way to achieve high positioning accuracy and obtain a trajectory close to the actual path in a common application scenario by smartphone without the use of a complicated algorithm. In the actual positioning process, a stable signal source can reduce the signal interference caused by environments. Bluetooth low energy has its own advantages in indoor positioning because it can be seen as a more stable signal source. In this study, we used smartphones to record the changing Bluetooth signals and used a basic nearest neighbor, weight centroid, and probability-based method, which we called an advanced weighted centroid method, to obtain position coordinates and the motion trajectory during the experiment. We used a weight centroid method based on least squares to solve the overdetermined problem. This can also be used to calculate the initial position of the advanced weight centroid. The advanced weighted centroid method introduced a Gaussian distribution to model the distribution of the signal. Translating a deterministic problem into a fuzzy probability problem aligns more with positioning facts and can achieve better results. Experimental results showed that the root-mean-square error (RMSE) of the dynamic positioning result obtained through the probabilistic method was within 1 m and had a more consistent trajectory. Moreover, the impact of the number of iBeacons on the positioning accuracy has been discussed, and a reference for iBeacon placement has been provided. In addition, an experiment was also conducted on the effect of signal transmission frequency on accuracy.

## 1. Introduction 

Location information plays an important role in the social economy and people’s lives. One study showed that humans spend almost 70% of their time indoors [1]. Therefore, obtaining an accurate indoor location has become especially important. The widespread use of smartphones and mobile internet have made real-time precise positioning possible, and this method is gradually being applied to various situations. For example, merchants use it to send advertising to customers nearby, and airport passengers can track their luggage in real time.

The common methods for indoor positioning mainly include nonradio technologies and wireless technologies. Nonradio technologies always include magnetic positioning, inertial measurements, and visual positioning. Wireless technologies include wireless fidelity (Wi-Fi), Bluetooth, and radio frequency identification (RFID)/near-field communication (NFC). Nonradio technologies like magnetic positioning [2] need to collect data in advance of making positioning calculations; however, because the magnetic field is susceptible to environmental interference, determining an initial position is difficult. Inertial navigation is prone to accumulation of errors, and trajectories can also drift [3]. Vision-based positioning [4] takes a large amount of computation time because it requires an image to be processed, and it is more sensitive to environmental changes. For example, a significant change in illumination can cause feature point matching to fail and thereby affect the positioning result. Therefore, we considered using wireless technologies for positioning. Our method did not require the collection of data in advance. The source of the signal was also a more stable Bluetooth low energy (BLE) source, and there was no accumulation of errors. This represents a viable improvement in positioning methods.

Currently, in the field of wireless positioning, it is common to obtain location information via methods including the global positioning system (GPS), RFID, ultrasonic technology, ultra-wideband (UWB) technology, ZigBee, Wi-Fi, and Bluetooth low energy [5,6,7]. Of these, GPS can obtain position results with high precision in outdoor environments. However, for indoor environments, building occlusion interferes with GPS signals and prevents location information from being acquired [8]. Furthermore, although RFID, ultrasonic, UWB, and other technologies have a high positioning accuracy, their deployment cost is high, and they cannot be applied widely to usual application environments. Thus, because of the convenient configurations and good positioning effects, Wi-Fi and Bluetooth are widely used in indoor positioning [9]. The signals they send can be received by smartphones, which commonly have built-in Wi-Fi and Bluetooth sensors. Smartphones, as an indispensable tool for modern people, can be a convenient choice for positioning in daily use.

Some indoor positioning methods focus on Wi-Fi, as it is now equipped in most indoor environments. However, in practical applications, Wi-Fi is susceptible to environmental changes, resulting in poor positioning accuracy. The development of BLE technology has attracted great interest in recent years, and it is considered by some researchers to be a more advantageous indoor signal source than Wi-Fi. Several studies have analyzed the signal characteristics of BLE and Wi-Fi and used the same positioning algorithm to compare the different positioning results obtained via different signal sources [10,11]. Compared to Wi-Fi, the iBeacon—a BLE implementation protocol from Apple [12]—has the characteristics of low power consumption, a smaller device size, better anti-inference systems, and easy arrangement. Therefore, BLE is suitable and effective for applications in the practice of indoor positioning [13]. 

Apple released the iBeacon standard in 2013, based on BLE 4.0. With the reduction in the cost of BLE devices over time, BLE is supported by almost all smartphone operating systems [14]. In addition, the Google Eddystone^TM^ open standard, launched in 2015, provides a new and rich broadcast format that supports multiple frames [15]. This promotes the rapid development of Bluetooth iBeacon platforms deployed and embedded in smart devices.

Both Wi-Fi and Bluetooth broadcast a signal comprising specifics encoded to the surroundings. The process of indoor positioning includes obtaining the received signal strength indication (RSSI) through a smartphone under dynamic conditions, obtaining path trajectories and smartphone coordinates via a certain algorithm. Several such methods have been proposed over the years. 

The nearest neighbor is a simple and applicable method that determines the nearest iBeacon by finding the maximum value of the received signal, and it considers the nearest iBeacon position as the position of the smartphone location [15]. However, the positioning results are poor, and with the expansion of the experimental area, the positioning accuracy declines significantly, especially when there is a small number of iBeacons. In other words, positioning accuracy is directly affected by iBeacon arrangement; therefore, achieving a higher accuracy requires a larger number of iBeacons. Furthermore, this positioning method is commonly used in real application scenarios. [16].

Weighted trilateration uses geometric relationships to determine where a smartphone is located. It obtains a location by calculating the distance to multiple iBeacons through RSSI and a signal attenuation model [17,18]. Of course, position can also be determined by the angles to iBeacons. Weight selection is usually determined according to the Euclidean distance [19]. Although this algorithm can theoretically obtain more accurate positioning results than nearest neighbor while ensuring low complexity, in reality, indoor signal propagation is often complicated because of the signal’s vulnerability to interference. There are often problems, such as the fact that multiple circles cannot be crossed at one point and become overdetermined, resulting in loss of positioning accuracy; this problem has been the focus of previous studies [15,20]. The fingerprint algorithm is another commonly used positioning algorithm with online and offline phases. The offline phase mainly collects data to build a fingerprint database. In the online phase, a user’s current position is determined via the real-time processing of the iBeacon or Wi-Fi fingerprint [18]. In general, the offline fingerprint database is matched with online positioning through deterministic algorithms [21] and probability-based algorithms [22]. The advantage of fingerprint positioning is that there is almost no need to know the coordinates of any iBeacons, and the positioning accuracy is relatively high. However, a high workload is required to establish the offline fingerprint database. In addition, this technique does not easily adapt to large changes in the environment. Positioning accuracy is excessively dependent on the accuracy of the fingerprint library. Therefore, several studies were conducted to solve these problems. Huang et al. [13] proposed combining Bluetooth and Wi-Fi to improve the acquisition accuracy of the fingerprint database. Zuo et al. [23] proposed reducing the workload of fingerprint database collection through interpolation.

Different positioning algorithms have their own application scenarios and conditions under which they are applicable, but few studies have used the probability distribution of RSSI itself for positioning. We were inspired by the probability algorithm used for fingerprint-database matching, which uses a Gaussian distribution to obtain the probability of the smartphone location [11]. In this technique, for each RSSI value received, the possible location of a smartphone is considered as a range rather than as coming from a specific point. Based on this idea, Hansson and Tufvesson [24] divided an experimental site into equal-sized grids and calculated the probability that the smartphone would be distributed in different grids. The grid with the highest probability was considered the location of the smartphone. They used Wi-Fi as the signal source, and the error obtained under static conditions was within 10 m. It is difficult to use this to meet the requirements of high-precision indoor positioning.

The goal of this study was to obtain the absolute two-dimensional location conveniently in a normal environment without harsh conditions. Therefore, because we do not always pay attention to the height of a mobile phone in indoor use, this study did not consider a three-dimensional situation, thus reducing the number of calculations required. Wi-Fi was no longer used as a signal source, instead we used a more stable and lower power-consuming BLE for positioning. We used the nearest neighbor algorithm as the basic positioning algorithm weight centroid, and we focused mainly on developing an advanced weighted centroid algorithm using probability distribution. Compared to previous probability-based methods, the least-squares method was used to solve the problem of overdetermination in the algorithm. We did not use dedicated signal receiving equipment. Our signal receiving device was a smartphone because it has become an indispensable tool for our daily use. The error obtained in the experiment was less than 1 m when using Bluetooth; as such, we were able to get high precision using a simple algorithm. We also avoided the labor of building a fingerprint database in the early stage of the algorithm, which is complex to implement. Therefore, this method strikes a good balance between the complexity of obtaining a position and ensuring its accuracy. This technique also has strong applicability and a good fault tolerance. Moreover, while our focus was on accuracy, we were also able to determine whether the positioning of the path was reasonable and the degree of agreement with the real path. We also discussed how the number of iBeacons affected positioning accuracy. We have explored whether the signal transmission interval influenced the positioning results. 

The remainder of the article is structured as follows. Section 2 details our positioning algorithm. In Section 3, we show the implementation of our experiments and the devices we used in the experiments. In Section 4, we demonstrate the positioning results using different algorithms, and we explore how the density of the iBeacon devices affected the accuracy of the experimental results. Finally, Section 5 presents our conclusions.

## 2. Methods

This section introduces the radio signal attenuation model and three positioning methods. The results of the positioning are given in Section 4.

### 2.1. Signal Characteristics and Radio Signal Attenuation Model

**Figure 1:**
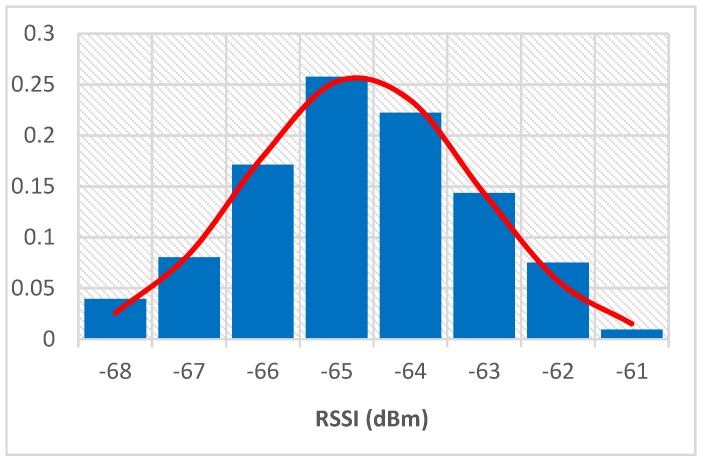
Signal value distribution characteristics accepted at 1.5 m. RSSI, received signal strength indication.

To determine the characteristics of RSSIs from beacons, we collected RSSIs through a smartphone at a certain distance from a beacon for 7 h. During this period, we randomly walked around the beacon to simulate the signal characteristics under real conditions. By counting the signal values, we obtained an RSSI distribution histogram and fitting curve, as shown in Figure 1, according to which the RSSI obtained at a fixed position fit well with a Gaussian distribution.

Several experiments have demonstrated a logarithmic relationship between signal strength and distance [20,25,26]. Bluetooth signal propagation is formulated using the following well-known radio signal attenuation equation:

(1) $$P_{L}\left(d\right)=P_{L}\left(d_{0}\right)+10nlog\left(\frac{d}{d_{0}}\right)+X_{\delta },$$

where $P_{L}\;\left(d\right)$ is the RSSI at distance *d* from the signal source; $P_{L}\;\left(d_{0}\right)$ is the RSSI when the value of $d_{0}$ is taken as reference distance; *n* represents the path loss exponent and is closely related to the surrounding environment [27]; and $X_{\delta }$ is a Gaussian random error factor with a mean of 0. However, we used a simplified formulation in real calculations based on Equation (1), with a reference distance is of 1 m; it is expressed as follows:

(2) P=A−10nlogd,

where *P* indicates the RSSI when it is at distance *d* from a signal source, and *A* indicates the signal strength 1 m from the source. The expression of the distance can be derived from Equation (2) as follows:

(3) $$d=10^{\frac{A-P}{10n}}.$$

### 2.2. Nearest Neighbor

This is the basic algorithm that we used in our experiments, given its advantages of simple operation, low complexity, and easy implementation. We were able to record a series of Bluetooth signal values using a smartphone and then sort the values of these signal strengths. The position of the iBeacon with the highest RSSI was considered our current location. The series of signal values can be given as follows:

$$R_{n,t}=\left\{r_{n},r_{n-1},r_{n-2},\cdots r_{1}\right\}$$

(4) $$S_{n}=\max\left(r_{i}\right),r_{i}\epsilon R_{n,t}$$

where $R_{n,t}$ is a dataset that collects signals transmitted by different iBeacons b at time *t*, and $S_{n}$ is the location of the iBeacon that emits the maximum RSSI.

However, as the experiment included asynchronous measurements, and the signal was unstable, the use of the nearest neighbor algorithm alone caused a large deviation between the previous and following steps. Moreover, in the experiment, we found that the last positioning point was in front of the device, while the next positioning point could return to a previous position, indicating that the path was backward. This was obviously inconsistent with the actual situation.

### 2.3. Weighted Centroid

**Figure 2:**
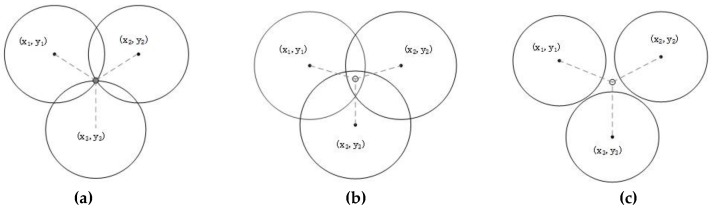
(**a**) Three circles intersect at one point under ideal conditions. (**b**) Three circles with coincident areas that do not intersect at one point. (**c**) Three circles with no intersecting areas.

In various indoor positioning algorithms, trilateration is a method that is simple and easy to implement. Theoretically, if the distance from the smartphone to three or more iBeacon nodes is known, the latter can be used as the center of the circles, and the distance to the smartphone would be the radius; these circles will intersect at a point. The common point at the intersection of these circles is the location we want, as shown in Figure 2a.

According to Equation (3), we can determine the length of the radius. The coordinates of a smartphone can be obtained by solving the following equation:

(5) $$r_{i}=\sqrt{{\left(x-x_{i}\right)}^{2}+{\left(y-y_{i}\right)}^{2}}.$$

However, because the signal is disturbed and attenuated when an obstacle is encountered—and owing to the multipath effect and measurement error of the iBeacon itself—these circles do not intersect at one point. Furthermore, if the part of the junction was an area (Figure 2b), or if there was no common area (Figure 2c), using this algorithm alone could not provide a convincing positioning result.

As we often obtained more than three signals in the experiment, we faced the problem of overdetermination, which can generally be solved using the least-squares method [3,28]. The least-squares (LS) method is a mathematical optimization technique that obtains the best match for the data by minimizing the sum of the squares of the errors. The objective function is constructed as follows:

(6) $$f_{i}\left(x,y\right)=r_{i}-\sqrt{{\left(x-x_{i}\right)}^{2}+{\left(y-y_{i}\right)}^{2}},$$

(7) $$\min\left(x,y\right)=\min\sum_{i=1}^{m}{\left[f_{i}\left(x,y\right)\right]}^{2},m\geq 3.$$

It is difficult to solve the nonlinear Equation (6) directly. Therefore, we transformed the problem into an optimization problem in Equation (7). Equation (7) could then be solved to obtain a more satisfactory result.

To simplify the calculations, we let $f_{i}\left(x,y\right)=0$ in Equation (6) and squared both sides to obtain

(8) $$x^{2}+y^{2}-2xx_{i}-2yy_{i}=d_{i}^{2}-x_{i}^{2}-n_{i}^{2}.$$

Next, we set $x^{2}+y^{2}=z$ to obtain the following equation:

(9) $$\begin{array}{c} z-2xx_{1}-2yy_{1}=d_{1}^{2}-x_{1}^{2}-n_{1}^{2},\\ z-2xx_{2}-2yy_{2}=d_{2}^{2}-x_{2}^{2}-n_{2}^{2},\\ \cdots \\ z-2xx_{m}-2yy_{m}=d_{m}^{2}-x_{m}^{2}-n_{m}^{2}. \end{array}$$

Thus, if we define the following:

(10) $$A=\left[\begin{array}{ccc} 1 & -2x_{1} & -2y_{1}\\ 1 & -2x_{2} & -2y_{2}\\ & \cdots & \\ 1 & -2x_{m} & -2y_{m} \end{array}\right],$$

(11) $$z=\left[\begin{array}{c} z\\ x\\ y \end{array}\right],$$

(12) $$\mathrm{and}\;b=\left[\begin{array}{c} d_{1}^{2}-x_{1}^{2}-n_{1}^{2}\\ d_{2}^{2}-x_{2}^{2}-n_{2}^{2}\\ \cdots \\ d_{m}^{2}-x_{m}^{2}-n_{m}^{2} \end{array}\right],$$

then Equation (9) can be written as follows:

(13) Az=b.

The LS technique can now be applied to calculate *z*. Therefore, the coordinates of target point (e, n) can be obtained as follows:

(14) $$z={\left(A^{T}A\right)}^{-1}A^{T}b.$$

The weighted centroid algorithm is used to estimate the coordinates of unknown points. The centroid is the point where the abscissa and ordinate are defined by the average of the surrounding N points. However, in practical applications, not all points have the same impact on the outcome. We usually use weighted methods to consider more relevant points for a larger proportion of the results. To ensure that higher RSSIs received higher weights and greater priority, we used Equation (15) to convert the RSSI unit to dBm.

(15) $$RSSIP_{i}=10^{RSSI_{i}/10},$$

where $RSSIP_{i}$ represents the transformed result in mW.

Each RSSI was weighted, and a set of smartphone coordinates was calculated using Equations (16) and (17), respectively, as shown below:

(16) $$w_{i}=\frac{RSSIP_{i}}{\sum PSSIP},$$

(17) $$Pos_{s}=\left(\sum_{i=1}^{N}w_{i}x_{i},\sum_{i=1}^{N}w_{i}y_{i}\right),$$

where $w_{i}$ is the weight, and $Pos_{s}$ indicates the coordinates of the smartphone.

### 2.4. Advanced Weighted Centroid

**Figure 3:**
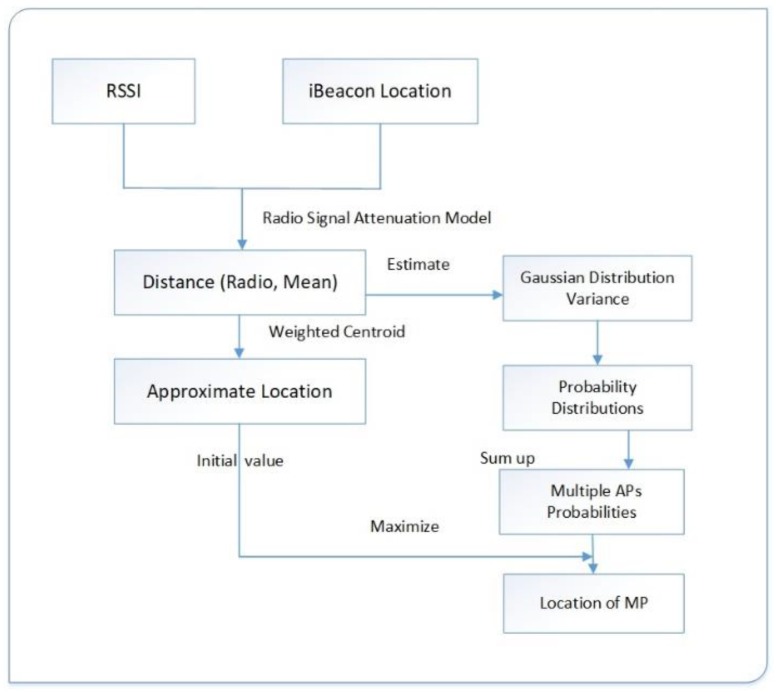
Process of implementing the advanced weighted centroid algorithm.

Similar to the weighted centroid algorithm, the advanced weighted centroid algorithm also processes RSSIs to obtain location coordinates. The difference is that we used a mathematical model to convert the original fixed position into the probability of being distributed at different locations. By superimposing the probability distribution around each iBeacon, the position obtained by the weighted centroid algorithm was taken as the initial value, and the location of the maximum value was considered to be the coordinates of the smartphone. The basic framework of the algorithm is shown in Figure 3.

**Figure 4:**
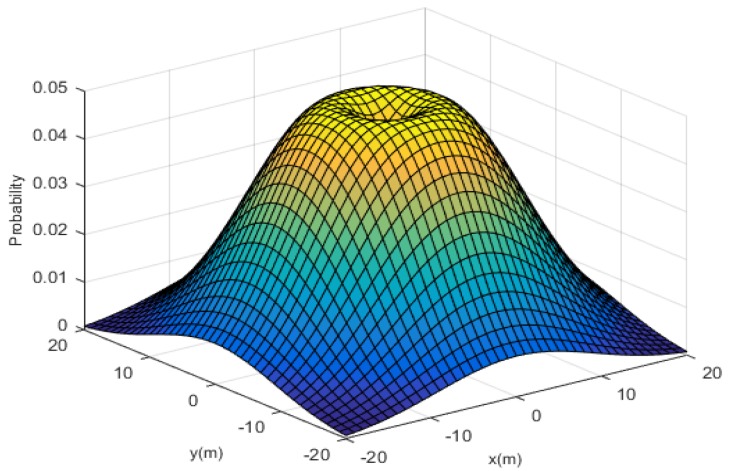
The possibility of a smartphone 5 m from the iBeacon being distributed at different locations in three dimensions.

The method we used introduced a Gaussian distribution based on the triangulation algorithm to improve the positioning accuracy. Through the above analysis, we already knew that, given an RSSI, a smartphone may not necessarily be located on the circumference, as calculated by Equation (4), but could in fact be located at any point in the experimental space. The probability of the smartphone being positioned at different distances from the signal source varies, with the probability that it exists on the circumference being highest. The probability of each of the different locations generally represents a Gaussian distribution. Therefore, we meshed the entire area with an interval, for example 0.1 m, and regarded every iBeacon as the center of each Gaussian distribution. Thus, we could obtain the probability density for smartphones on different grids. As shown in Figure 4, different iBeacons generate different probability distribution maps; thus, every grid has multiple probability density values, and the number of these values is equal to the number of iBeacons [29]. By summing these values, the highest grid can be considered the current location of the smartphone. 

The Gaussian distribution represented in Equation (18) has two important parameters: mathematical expectation representing the mean value μ and a standard deviation representing the degree of dispersion σ.

(18) $$f\left(x\right)=\frac{1}{\sqrt{2\pi }\sigma }exp^{\left(-\frac{{\left(x-\mu \right)}^{2}}{2\sigma^{2}}\right)}.$$

As shown in Figure 4, the highest point in the image, shaped like a ring, represents the circle shown in Figure 2 and is the most likely location of the point. Based on this idea, the expectation can be obtained through Equation (3), and the distance to the circle can be expressed as below:

(19) $$\Delta d_{k}=\sqrt{{\left(x\left(k\right)-x_{c}\right)}^{2}+{\left(y\left(k\right)-y_{c}\right)}^{2}}-d,$$

where $\left(x_{c},y_{c}\right)$ is the center of the circle and the coordinate of the iBeacon, and $\Delta d_{k}$ indicates the distance between each grid point and circumference with radius d. This formula represents the degree of deviation of each grid point from the average level. It can be analogized to x−μ in Equation (18).

**Figure 5:**
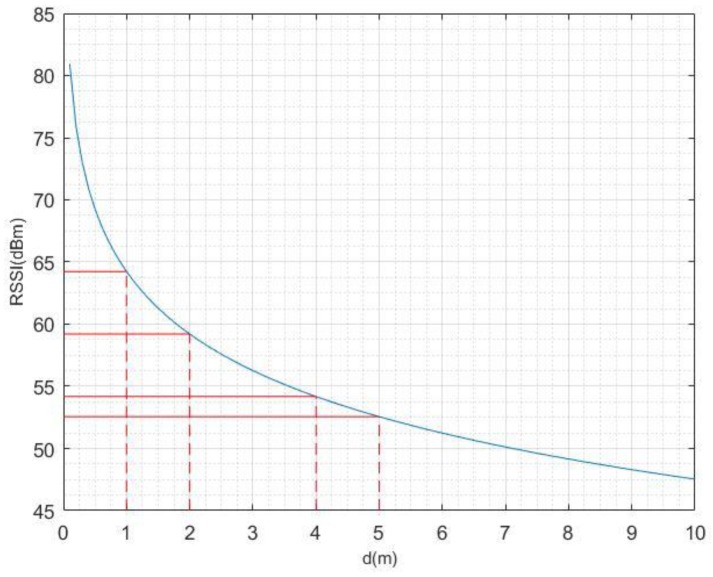
Changes in RSSI as the distance from the iBeacon increases.

The probability that a smartphone is distributed at different positions can be expressed as follows:

(20) $$p_{ij}\left(k\right)=\frac{1}{\sqrt{2\pi \sigma^{2}}}\cdot e^{\frac{-\Delta d_{ij}{\left(k\right)}^{2}}{2\sigma {\left(d\right)}^{2}}},$$

where σ is the standard deviation for the Gaussian distribution. The standard deviation reflects the degree of dispersion between individuals within a group. In Figure 5, the curve shows the shape of the concave function, indicating that the same change in distance corresponds to different changes in RSSI. In our algorithm, every smartphone corresponds to several iBeacons, each of which has a corresponding σ.

From Equation (20), we can see that path losses *n* and A indicate that the RSSI at 1 m had a significant influence on result P. Although σ may not have the same effect on the result, it is an important parameter in this algorithm. The standard deviation changed with the distance; the farther from the iBeacon, the larger the variance and the larger the standard deviation were [3]. Equation (21), shown below, was used to calculate σ:

(21) $$\sigma \left(d\right)=\alpha +\frac{d}{\beta },$$

where α and β are constant and obtained empirically. In the actual experiment, the equation was adjusted; we let α = 5 and β = 3.

To obtain the final probability of each grid, we needed to account for the probabilities from each measurement. The operation is as follows:

(22) $$g_{ij}=\sum_{k=1}^{N}p_{ij}\left(k\right).$$

**Figure 6:**
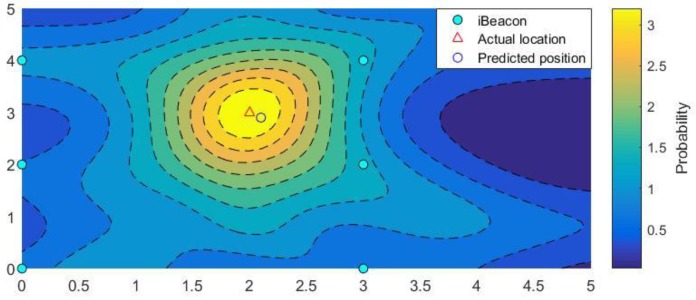
Schematic of the probability distribution obtained via computer simulation after the addition.

The position coordinates at which the smartphone is most likely to be located are where $g_{ij}$ is the maximum. Figure 6 shows the probability distribution contour map considering the observations of multiple iBeacons within the test site. This schematic was drawn mainly from the results obtained through computer simulation experiments. It can be seen intuitively that the closer the simulation approached the real position, the higher the probability became. The simulation results obtained through this method were very close to the true position.

In the actual calculation, we did not divide the grid; instead, the maximum value was obtained by optimizing an objective function, Equation (22), equivalent to finding the grid with the smallest value. We used the quasi-Newton algorithm, which is well-recognized and effective [30], as our optimization method. The initial value of the optimization was the position coordinate obtained via the weighted centroid method.

## 3. Experiment

In this section, we present the implementation of our experiments, the devices used, and how data were processed.

### 3.1. Device and Software

**Figure 7:**
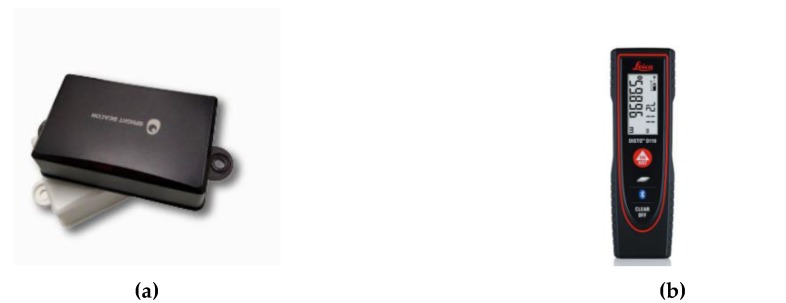
Experimental apparatus. (**a**) Max iBeacon from Bright iBeacon. (**b**) DISTO D110 laser distance meter from Leica Geosystems.

The iBeacon used (Figure 7a) was part of the Max iBeacon series from Bright iBeacon (Bright Beacon in Chongqing, China). This product has a very low energy consumption and can run for two to three years on two AA batteries. The nRF51822 chips [31] (NORDIC in Oslo, Norway) built into these devices are ideally suited for BLE and 2.4 GHz ultra-low-power wireless applications. We set the transmission time interval to 0.1 s to help us obtain more data and for ease of data processing.

Based on the results of previous experiments [3,23], we have acquired considerable prior knowledge about laying iBeacons. We investigated the experimental site in advance to determine the deployment of the iBeacons in the experiment. The iBeacons were placed in a rectangle and distributed evenly in the network at 1.2 m above the ground. This way, we were able to disregard the results of different distribution characteristics. When we first started the experiment, we placed more iBeacons for extra observations to allow us to explore how the number of iBeacons influenced the accuracy of the results by continuously reducing the number of iBeacons placed. We recorded the signals sent by iBeacons through our own Android application on an OPPO N1 smartphone (OPPO in Dongguan, Guangdong, China) and an Honor 8 smartphone (HUAWEI in Shenzhen, Guangdong, China) running the Android 7 operating system. 

As our positioning was not static, the true path coordinates were known before precision calculations. The distance to the two coordinate axes was measured, based on the previously established coordinate system, using the laser range finder on continuous trajectories. We used the DISTO D110 from Leica Geosystems (Leica in Wetzlar, Hessen, Germany), which has an accuracy of 0.0015 m (as shown in Figure 7b), in our experiment.

### 3.2. Experimental Sites

Our experimental sites were an 18.97×12.76 m conference room and a 6.64×5.63 m office. During the experiment, some people walked around randomly to simulate positioning in a real scene. 

**Figure 8:**
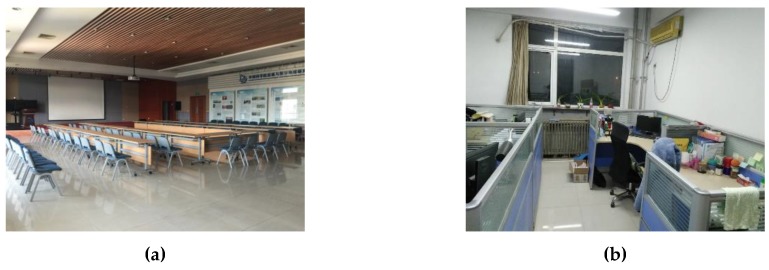
Experimental sites. (**a**) Conference room. (**b**) Open office.

**Figure 9:**
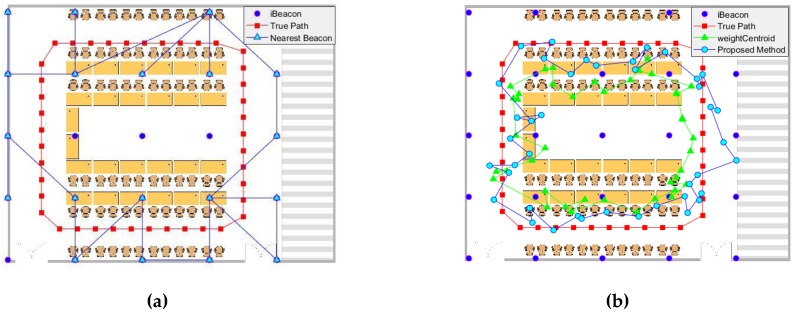
Distribution of iBeacons and the map of trajectories obtained by the (**a**) nearest neighbor and (**b**) advanced weighted centroid methods in a conference room.

The conference room was an open room with some tables and chairs that were placed as in Figure 8a. We placed the table in the center of the conference room. This allowed the tester to walk along a looping path around the desks. In this experimental scenario, we used a total of 25 iBeacons, and they were placed in a 5×5 grid at 3 m intervals. The position of the iBeacon is shown in Figure 9. The way we arranged iBeacons was based on experience in previous experiments. It has been found that placement like this can excellently avoid the situation of three or more circles having no intersection. The route we planned was a closed rectangular route surrounded by iBeacons. In this way, it is convenient to get the real path during the experiment, and the positioning result can be better displayed. 

**Figure 10:**
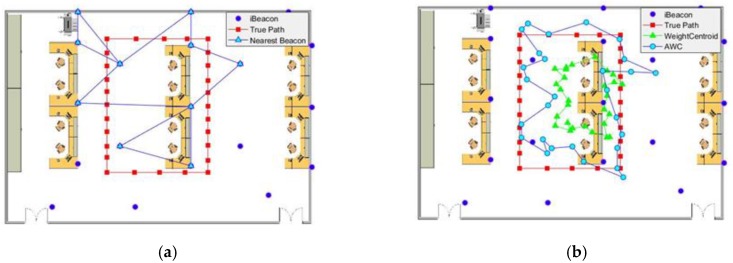
The map of trajectories obtained by the (**a**) nearest neighbor and (**b**) advanced weighted centroid methods in an office.

The office was a small room containing several desks and other items. The iBeacon placement was similar to the previous experimental scene. As this room was smaller, we used 19 iBeacons and placed them in a rectangular-like grid. The arrangement of more redundant iBeacons facilitated the subsequent study of the relationship between the number of iBeacons and positioning accuracy. As some desks in the office could not be moved, we placed the iBeacon on the partition of the desk. The spacing between each iBeacon was approximately 2 m. The positions of the iBeacon are shown in Figure 10. We also designed a loop motion trajectory in the aisles between the tables. This route mainly used the existing aisles in the office. This was a good way to simulate the route people would walk in the office.

### 3.3. Data Processing

Before the experiment started, we needed to measure the RSSI at 1 m and obtain the path loss value at the experimental sites. The measurement was performed continuously for 30 s at a distance of 1 m from the iBeacon, and the average value was taken as the value of A in Equation (3). The path loss values in the experimental scenario were deduced using the RSSI at different preknown distances, and LS regression was used to obtain the path loss value.

We set the iBeacon transmit power interval to 0.1 s and averaged 10 values to reduce the volatility of the environment and system itself. Thus, our position was updated every second, which was in line with human walking habits.

We entered the pre-processed data into the model to obtain the final positioning results. This result was verified with the accuracy of the coordinates obtained using a laser range finder. Both accuracy verification and picture drawing were performed using MATLAB (MathWorks in Natick, MA, USA).

## 4. Results and Discussion

In this section, we discuss the experimental results including the trajectory and accuracy analyses obtained through the different algorithms. In addition, we showed how the density of iBeacons influenced the positioning accuracy.

### 4.1. Positioning Trajectory Analysis

Figure 9 shows the positioning results obtained in the conference room. 

According to Figure 9a, the tester completed the planned circular path at a generally uniform speed; this path is shown as the red closed loop in the figure. It took almost 49 s to complete the rectangular path. In this figure, the blue dots represent the placement of the iBeacons, and the blue line is the trajectory calculated using the nearest neighbor method. This method achieved an RMSE of 2.132 m in the conference room. In addition to the low positioning accuracy, we determined that because the nearest iBeacon was regarded as the current position of the smartphones, the trajectory obtained by the nearest neighbor method was completely different from the actual trajectory. In real-time positioning, the positioning point may not change continuously, and a transition would be observed between points. Although this algorithm was simple and took little time, it had a low accuracy and resulted in inaccurate paths. This method can be used in situations where the accuracy requirements are low and there is a need for the position to be determined quickly. For higher positioning accuracy, a different algorithm must be considered.

In Figure 9b, the green path shows the use of a simple triangular weighting algorithm. The position of the triangle on the green path was also the initial position that we optimized. As shown, this position was not far from the real position, and calculations from this position greatly shortened the convergence time. The cyan curve represents the results obtained using the advanced weighted centroid algorithm. We already knew that using the nearest neighbor method could not produce a result close to the actual trajectory. This method had almost the same trend as the real path, and the trajectory was closer to the real situation. In addition, this method also achieved good positioning accuracy, with an RMSE of 0.9184 m. The accuracy was greatly improved relative to the nearest neighbor method. In addition, the coordinates we calculated changed as we moved.

**Figure 11:**
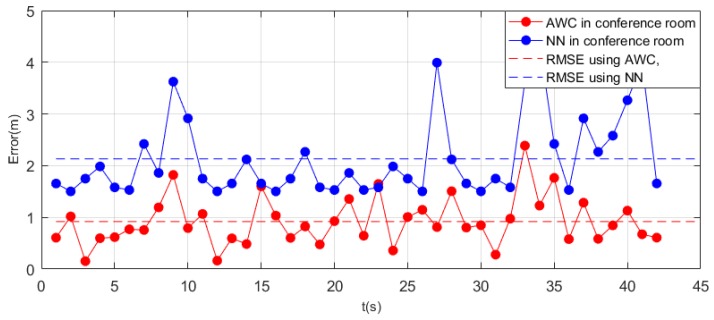
Error of different algorithms in a conference room. AWC, advanced weighted centroid method; NN, nearest neighbor method. RMSE, root-mean-square error.

As shown in Figure 10, a smaller office was used as the experimental scene. It took 31 s to complete the entire path, and 19 iBeacons were used. As shown in Figure 11, the trajectory obtained in the office by the nearest neighbor algorithm was more unreasonable because of the reduction in the number of iBeacons. In the lower part of the figure, the nearest neighbor algorithm produced a path re-entry phenomenon because of the fluctuation of the data. Thus, this method was sensitive to abnormal values. Although the RMSE was reduced because of the increased iBeacon density, this was not a reliable algorithm and did not have high precision.

Using the advanced weighted centroid method, we reached the same conclusion as in the conference room: this method matched the actual path more closely than the nearest neighbor method did. As shown at the bottom of the image, the path obtained by the advanced weighted centroid did not return to the right path. This shows that our approach reduced the sensitivity to inaccurate data. Therefore, this algorithm can provide stable and high-precision positioning services. Thus, in general, the use of the advanced weighted centroid method had better fitting effects in both a conference room and an office.

### 4.2. Positioning Accuracy Analysis

Next, we conducted a quantitative analysis of the errors of the various methods. The error was mainly derived from the signal interference during the experiment; the iBeacon itself was not stable. It was also impossible to produce an algorithm capable of obtaining the true value, and there were inevitable systematic errors.

Figure 11 shows how the error between the real position and the points we calculated in the conference room changed over time. The RMSE calculated in a conference room using the advanced weighted centroid (AWC) method was 0.9184 m, which was considerably less than the error of 2.1326 obtained by the nearest neighbor algorithm (NN). 

However, it is apparent from the figure that almost all errors obtained at each moment using the improved method were less than the nearest neighbor method. This method obtained a more uniform error distribution, and there was no point at which the error far exceeded the RMSE. The figure shows that the error of the nearest neighbor method varied greatly, and the trajectory obtained by the nearest neighbor differed greatly from the real trajectory.

**Figure 12:**
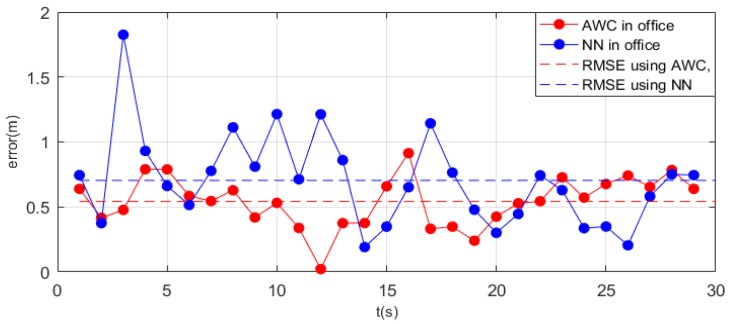
The error of different algorithms in an office. AWC, advanced weighted centroid; NN, nearest neighbor; RMSE, root-mean-square error.

The situation was also evident in the office. Figure 12 shows how the error in the office changed over time. Overall, the RMSE of the nearest neighbor algorithm was still higher than that of the advanced weighted centroid algorithm. In the first 13 s, the error of the nearest neighbor was significantly higher than that of the advanced weighted centroid and showed strong volatility. However, in the last 16 s, the errors of the two algorithms were very close, and even the accuracy of the nearest neighbor algorithm exceeded that of our algorithm. In summary, the error of nearest neighbor was first less than 1 m, and then it reached an acceptable error range. Errors were also highly likely to be random. Moreover, in the latter half of the experiment, some iBeacons were simply placed near the last arriving point; thus, the positioning of these iBeacons greatly improved the positioning accuracy. Thus, it is again clarified that the nearest neighbor method is heavily dependent on the arrangement of iBeacons.

### 4.3. Effect of the Number of iBeacons on Accuracy

**Figure 13:**
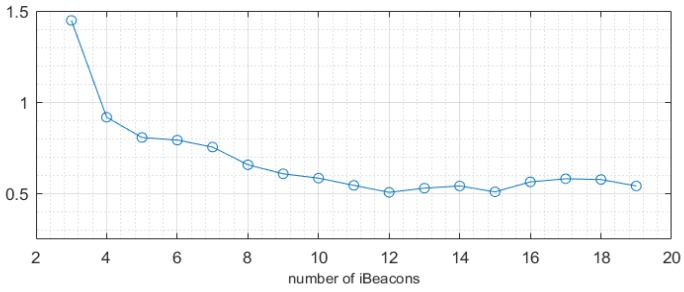
Variation in error given the number of iBeacons in the conference room.

To estimate the effect of the number of iBeacons on the result, we used a different number of iBeacons in our two experiment rooms using an advanced weighted centroid. Figure 13 shows the test result in the conference room. From the curve in the figure, we can see that the positioning accuracy increased as the number of iBeacons increased. When Bluetooth is used in actual positioning, a few iBeacons will result in very unreasonable positioning results. This is caused by a lack of other reference values to reduce the contingency of a few measurements. Therefore, within a certain range, the increase of the iBeacons quickly reduces the average RMSE error. This can explain that the addition of repeated observations helps improve accuracy. However, as the number of iBeacons increases, the improvement in positioning accuracy becomes less obvious, and the error is more stable. This indicates that continuing to increase the number of iBeacons does not necessarily reduce system and other errors.

**Figure 14:**
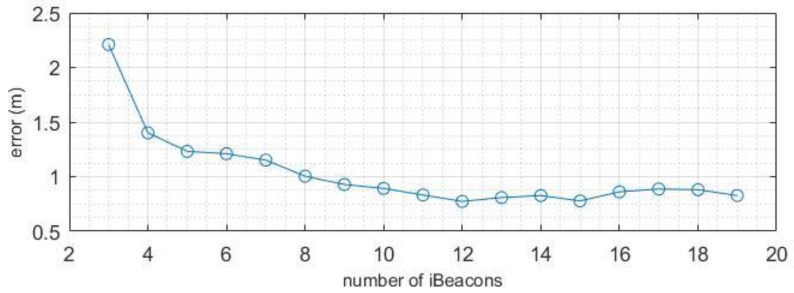
Variation in error given the number of iBeacons in the office.

As the office was a smaller room, the impact of the number of iBeacons was more evident. Figure 14 shows that the downward trend in the curve in the office was similar to the scenario in the conference room. In addition, the error decreased quickly between 3–8 iBeacons. When the number of iBeacons was greater than eight, the improvement in accuracy was less evident. The curve appeared to fluctuate, and the accuracy increased. After the number of iBeacons reached a certain level, we could not guarantee that the redundant observations were sufficiently accurate. If the measured values were inaccurate, the error will increase slightly. In addition, we found that in the first experimental scenario, after approximately 17 iBeacons were placed, the increase in accuracy was less noticeable. In the office, the improvement in accuracy was limited when more than eight iBeacons were used. This explains that the number of iBeacons required depends considerably on the area of the site.

This experiment indicates that, in an actual application of Bluetooth positioning, a priori experiments can be performed to decide the number of iBeacons used. In this way, we can obtain a high positioning accuracy without the economic loss caused when redundant iBeacons are placed.

We also considered the effect of different layout densities on the accuracy of the experiment; therefore, we considered both the size of the experimental site and the number of iBeacons.

**Figure 15:**
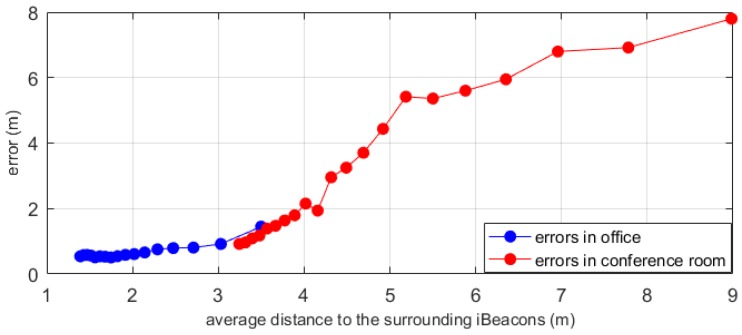
The effect of beacon placement density on positioning accuracy.

The average distance between two adjacent iBeacons reflects the density of beacon placement. The greater the average distance, the lower the density. The changes in density and accuracy are shown in Figure 15. In general, the greater the density of beacon placement, the higher the positioning accuracy. When the average distance between two adjacent iBeacon is 3–4 m, the error values in the two positioning environments almost coincide. This explains why different positioning environments have negligible effects on the accuracy. The layout density of the beacon affects the accuracy of the positioning within a certain range. When the distance between the beacons is small, that is, after the indoor beacon density reaches a certain level, it is difficult to improve the accuracy. This was also observed in our previous experiment. Therefore, when planning the layout density of the beacons, the size of the scene should be considered.

### 4.4. Effect of Different Emission Frequencies on Accuracy

**Figure 16:**
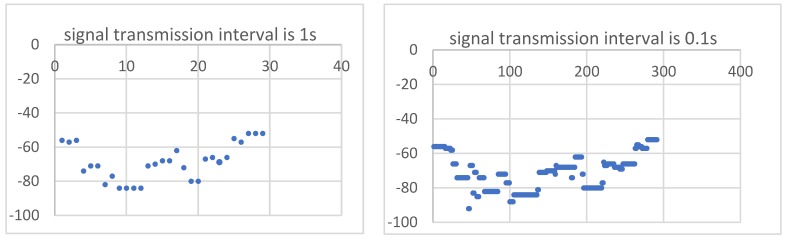
RSSI distributions of different signal transmission frequencies.

To study the effect of the time interval of signal transmission on the positioning accuracy, we set the signal transmission interval of the iBeacons to 0.1 s and 1 s. We calculated the statistics on the distribution of signals. Figure 16 shows the RSSI distributions of different signal transmission frequencies. For an identical iBeacon, the signal distribution is approximately the same even if the transmitted signal frequency is different during the positioning process.

**Figure 17:**
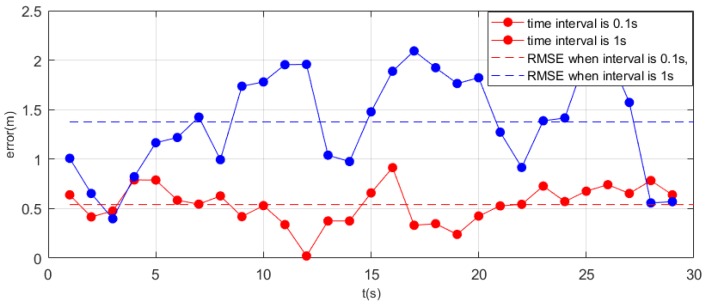
Different errors caused by different signal transmission time intervals.

In this experiment, we mainly used a transmission frequency of 0.1 s. In theory, averaging more measured values can reduce accidental errors. For experiments with a transmission time interval of 0.1 s, we averaged 10 values to obtain the signal value over 1 s. From Figure 17, it can be seen that the overall error after averaging was significantly less than that for the signal transmitted every 1 s. This also shows that when the time interval is 0.1 s, averaging the values in 1 s can effectively avoid errors caused by signal instability and signal occlusion.

## 5. Conclusions

This study used three positioning algorithms: nearest neighbor, weight centroid, and advanced weighted centroid. We switched the signal source from Wi-Fi to BLE, which is stable and has a lower power consumption. The nearest neighbor algorithm is relatively simple, but its role in practical applications cannot be ignored. This allows quick accuracy positioning without the need for high precision. We used this as a basic method throughout our experiment. We added the least-squares method to solve the problem of overdetermination in the weight centroid. Further, we introduced the advanced weighted centroid method, which treats the inaccurate results obtained by the centroid algorithm as the optimal initial value. Furthermore, our experiment found that the probability of a smartphone being distributed around the location of the iBeacon exhibited a Gaussian distribution. In this manner, we obtained the probability that smartphones would be distributed at different locations for multiple iBeacons. By summing the values in each grid, we found the maximum value, and the location of the grid was taken as the location of the smartphone being requested. This approach did not use signal strength to estimate the distance; instead, it treated all locations around an iBeacon as possible points of distribution. This reduced the error caused by the environment to a certain extent. Compared with the fingerprint method we used in reference [23], we avoided the trouble of building a fingerprint library in the early stages of the experiment and found it easier to start positioning. We could avoid the problem of re-establishing the fingerprint database once the arrangement of indoor facilities changed. We also explored the particle filtering algorithms. Based on the work of reference [3], we improved the average positioning accuracy and obtained accurate trajectory information in a manner that did not require the use of the inertial measurement unit in a mobile phone, thereby simplifying the positioning process. There is no need to perform weight updates and iterative operations on thousands of particles. The time complexity of the algorithm was also lower.

We designed experiments to verify the localization effect of these algorithms in practice and obtained substantially similar experimental results in two different scenarios. In both scenarios, the RMSE of the advanced weighted centroid was less than one meter. The average accuracy was higher than the nearest neighbor algorithm; moreover, the error distribution between the position obtained by this method and the real value was uniform; thus, a trajectory better than that provided by the nearest iBeacon can be obtained. 

To explore how the number of iBeacons affected the experimental results, we gradually reduced the number of iBeacons used. The results showed that the number of iBeacons influenced the accuracy of the experiment considerably for a certain range of the number of iBeacons. However, after reaching a certain value, the accuracy did not improve any further. In addition, we found that the threshold of the number of iBeacons was related to the size of the experimental area. Experimenting on the application site in advance enables higher positioning accuracy using as few iBeacons as possible. Furthermore, we comprehensively considered the number of iBeacons and the area of the room in two different experimental environments. It is evident that the positioning accuracy is significantly improved with the increase of average distance between adjacent iBeacons within a certain range. However, after reaching a certain value, the accuracy no longer rises significantly. And when the layout density is similar in different rooms, the errors obtained are also very close. In addition, we also discussed the effects of different signal transmission frequencies on different positioning accuracies.

All instruments used in the study were ordinary instruments, and no special equipment was used. Furthermore, there were no great expenses. The experimental settings were the work and meeting environments used in daily life, without special modifications. Thus, we believe that our method can meet indoor positioning needs under normal scenarios, has wide applicability, and is portable.
